# Surveys of clinician and patient attitudes to an add‐on for *in vitro* fertilisation

**DOI:** 10.1111/ajo.13576

**Published:** 2022-06-21

**Authors:** Karyn Anderson, Michelle Blaxall, Sarah Lensen, Cindy Farquhar

**Affiliations:** ^1^ Department of Obstetrics and Gynaecology University of Auckland Auckland New Zealand; ^2^ Department of Obstetrics and Gynaecology University of Melbourne Melbourne Victoria Australia; ^3^ Fertility Plus Clinic, Auckland District Health Board Auckland New Zealand

**Keywords:** *in vitro* fertilisation, survey, add‐ons, adjuvant therapy

## Abstract

**Background:**

Add‐ons at the time of *in vitro* fertilisation (IVF) have become commonplace, despite a general lack of evidence that they are effective and safe. The ‘Colorado Protocol’ is a commonly used add‐on consisting of aspirin, steroid and an antibiotic. Before commencing planning for a clinical trial evaluating the Colorado Protocol, researchers and funders need evidence that the Colorado Protocol is being prescribed, and to be assured that sufficient numbers of participants can be recruited for a clinical trial.

**Aims:**

To survey fertility clinicians and patients on attitudes toward use of add‐ons during IVF, willingness of patients to be randomly assigned to an add‐on trial treatment or placebo, and what would be the clinically meaningful outcomes, using the Colorado Protocol as a test case.

**Materials and methods:**

Two online surveys were conducted: clinicians from fertility clinics across the United Kingdom, Australia, and New Zealand; and patients from Auckland‐based clinics and NZ patient support groups.

**Results:**

Of 58 clinicians, 44 (75%) had recommended an add‐on within the preceding year. Thirty‐nine (67%) clinicians were aware of the Colorado Protocol, with 17 (29%) having recommended it within the preceding year. Of the 289 patients, 80% indicated willingness to take trial medications during IVF, and 68% were willing to be randomly assigned to the placebo arm of a trial. The median perceived minimum clinically important difference in live births in both samples was 5%.

**Conclusions:**

A future trial of this add‐on in IVF would be supported by patients in the context of the New Zealand fertility healthcare system.

## INTRODUCTION

Adjuvant therapies used in addition to standard *in vitro* fertilisation (IVF) protocols, often referred to as ‘add‐ons’, are offered in practice to fertility service patients. However, a review conducted by the Human Fertilisation and Embryology Authority (HFEA) concluded that not a single add‐on was sufficiently supported by high‐quality randomised controlled trial (RCT) evidence, and therefore none are currently recommended.^1^, ^2^

One add‐on, often referred to as the ‘Colorado Protocol’, consists of a combination of aspirin, steroid (usually prednisone), and an antibiotic (amoxicillin clavulanate or doxycycline). This combination therapy is offered in fertility clinics in the United Kingdom (UK), Australia and New Zealand, particularly to patients who have had previous IVF cycle failure. This add‐on protocol is the subject of a future RCT proposed by the authors to determine effectiveness in women with recurrent implantation failure (RIF).

Failure to enrol participants into a clinical trial is a common concern leading to underpowered studies, and delayed study completion. This is a particular concern for fertility trials, where large sample sizes are required to detect with statistical significance modest differences in live birth rate (LBR).^3^ A recent review of published fertility RCTs found few were in fact large enough to detect such improvements in LBRs.^4^ The possibility of being assigned to the placebo arm may lead to poor recruitment in clinical trials^5^ and has been reported as a particular concern for fertility patients.^6^

With these concerns in mind, we surveyed both fertility clinicians and patients with the following aims: to understand current use of and attitudes toward this add‐on in IVF; to assess the willingness of IVF patients to be randomised to either the active treatment or the placebo group in a randomised trial; and to assess for both groups what would be a clinically meaningful difference in live birth outcomes.

## MATERIALS AND METHODS

### Eligibility and recruitment

Two anonymous surveys were conducted. One survey recruited practising fertility clinicians at clinics based in the UK, Australia, and New Zealand. Clinicians were recruited by emailed survey invitation addressed to the Medical Directors of fertility clinics throughout Australia (84 clinics), New Zealand (seven clinics) and the UK (123 clinics), as identified through the Australian and New Zealand Assisted Reproduction Database (ANZARD) or the HFEA of the UK. Medical Directors were asked to reply to the research team to indicate the number of staff they had distributed the survey to, in order for a survey response rate to be calculated.

The second survey was of IVF patients in New Zealand only. Women who had undertaken a previous embryo transfer procedure, either as part of an IVF cycle (including intracytoplasmic sperm injection), or as a frozen embryo transfer, were invited. Women who had not had an embryo transfer previously; or those who indicated they had used or been a surrogate, had undergone IVF for oocyte donation, or had undertaken oocyte preservation, were excluded. The survey invitation was distributed by email to women who had undergone an embryo transfer procedure in the preceding 24 months at two Auckland fertility clinics, which provide both public and private care, and on the social media platforms of these clinics. Additional social media and websites posts were made from relevant organisations, such as Fertility New Zealand Charitable Trust (FNZ), the largest information, support, and patient advocacy group in New Zealand. The survey was open from 8 July to 10 September 2021.

In order to avoid influencing a participant's decision to take part, the invitations, and introductions to the surveys, did not specifically mention the Colorado Protocol.

### Survey design

Both surveys used Qualtrics^©^, and consisted of 20 questions, taking between five and ten minutes to complete (Appendix S1). Most questions were asked as multiple choice, with some allowing free text responses under ‘other’. The clinician survey was pilot‐tested with a small number of clinicians, and the patient survey tested with a small number of fertility patients at one of the Auckland fertility clinics, and reviewed by FNZ.

Clinicians were asked to indicate their frequency of recommending the Colorado Protocol, or its constituent medications, to patients, and their definition of RIF. Conditional logic was used to step IVF patients through a series of questions about their willingness to participate in a clinical trial of the combination of three medications, and if the clinical trial was placebo‐controlled. Reasons for maybe and no responses were collected.

An average IVF cycle LBR of 25% was provided, and both patients and clinicians were asked to indicate the minimum absolute improvement in LBR that would justify the use of three additional tablet medications, as per the Colorado Protocol (eg 5%, making a 30% LBR). The clinicians' survey allowed free text entry of their nominated value, while IVF patients were provided the choice of 1%, 3%, 5%, 8%, or 10% improvement. The survey also collected some demographic data, but respondents could select a ‘prefer not to say’ option.

### Statistical analysis

Due to the low number of responses, data analysis was restricted to descriptive statistics of the whole survey cohort, and statistical testing was not performed. When survey respondents selected ‘other’ in a multiple‐choice question, where possible, their free text answers were recorded as belonging to one of the listed options; however, additional categories were developed to group some responses. Percentages were calculated for all responses excluding missing data for each question.

### Ethics approval

These feasibility studies received ethics approval from the Auckland Health Research Ethics Committee (AH22123).

## RESULTS

Overall, 336 email invitations were sent to fertility clinic directors, with no clinic directors replying to indicate the number of staff they had distributed the survey to, precluding the calculation of a response rate. A total of 71 responses were received. Of these, 58 eligible responses were analysed, after removing responses from clinicians who did not complete any survey questions beyond acknowledging they had read the participant information (*n* = 8), or were ineligible due to not currently providing clinical fertility care (*n* = 5). Disproportionally fewer responses were received from UK‐based clinicians (Table 1).

**Table 1:** Characteristics of clinician survey respondents

	(*N* = 58) *n* (%)
Country of clinical practice	
New Zealand	23 (39.7)
Australia	14 (24.1)
United Kingdom	15 (25.9)
Missing	6
Clinical role	
Doctor	46 (79.3)
Nurse	9 (15.5)
Donor co‐ordinator	1 (1.7)
Missing	2
Prioritised ethnicity†	
Māori	1 (1.7)
Asian	8 (13.8)
Middle Eastern/ Latin American/ African	1 (1.7)
European	40 (69.0)
Prefer not to say	2 (3.4)
Missing	5

^†^ Self‐identified ethnicity prioritised according to the Ethnicity NZ Standard Classification V2.1.0 (Ministry of Health, 2017).

A total of 420 responses to the survey of IVF patients were received. After removing responses from women who did not complete any survey questions beyond acknowledging they had read the participant information (*n* = 80) or those who were ineligible (had not had a previous embryo transfer, *n* = 39; did not indicate how many embryo transfers they had, *n* = 12), the remaining 289 eligible responses were analysed. The ethnicity of women completing the survey was broadly representative of the New Zealand population (Table 2). Women had undergone an average of 2.7 embryo transfer treatments.

**Table 2:** Characteristics of *in vitro* fertilisation patient survey respondents

	(*N* = 289) *n* (%)
Previous embryo transfers	
1	101 (34.9)
2	59 (20.4)
3	53 (18.3)
4	28 (9.7)
5	19 (6.6)
6	8 (2.8)
More than 6	21 (7.3)
Prioritised ethnicity†	
Māori	22 (7.6)
Pacifica	9 (3.1)
Asian	56 (19.4)
Middle Eastern/ Latin American/ African	8 (2.8)
New Zealand European	117 (40.5)
Other European	25 (8.7)
Prefer not to say	7 (2.4)
Missing	42

^†^ Self‐identified ethnicity prioritised according to the Ethnicity NZ Standard Classification V2.1.0 (Ministry of Health, 2017).

### Clinicians' use of the Colorado Protocol and other IVF add‐ons

When provided with a list of add‐ons, 75.9% (44/58) of clinicians indicated they had recommended one or more within the past 12 months. Sixty‐seven percent (39/58) were aware of the Colorado Protocol, with 29.3% (17/58) having recommended or prescribed it within the preceding 12 months. Among clinicians, 58.8% had used the Colorado Protocol infrequently (1–5 times), 35.3% 5–20 times, and 5.9% 20 or more times.

The most common indication for the use of the Colorado Protocol was RIF (25.0%), although definitions of RIF varied, ranging from 2–5 embryo transfers without achieving a pregnancy. Other indications for the Colorado Protocol included patient request (17.9%), high natural killer cell count (8.3%), and recurrent pregnancy loss (8.3%). From 58 definitions of RIF offered by clinicians, 35 described three embryo transfers, with 16 of these premising this with embryos being of ‘good quality’.

### Willingness to participate among IVF patients

The burden of the Colorado Protocol (number of medications and/or length of intervention) would be, or might be, acceptable to most women having previously undergone IVF (three‐medication trial condition was acceptable to 78.2%; 12 weeks of medication condition was acceptable to 74.1%) (Table 3). The most common reason for hesitancy in willingness to participate, or declining participation in a trial of the Colorado Protocol, was concerns regarding the safety of medications in pregnancy (75.1%); with other reasons including too many medications (9.7%); a desire for more information (8.0%); excessive duration of medication (5.3%); not planning further treatment (4.2%); allergy to one of the medications (4.0%); and uncertainties of research (4.0%) (Table S1). Additionally, 8.3% of fertility patients surveyed indicated they had an allergy to penicillin.

**Table 3:** Agreement to participate in a clinical trial under varying conditions among IVF patients

	(*n* = 289) *n* (%)
Combination of 3 medications without placebo arm	
Yes	137 (47.4)
Maybe	89 (30.8)
No	35 (12.1)
Missing	28
Combination of 3 medications with placebo arm	
Yes	141 (48.8)
Maybe	58 (20.1)
No	60 (20.8)
Missing	30
Medication taken to 12 weeks of pregnancy without placebo arm	
Yes	143 (49.5)
Maybe	71 (24.6)
No	42 (14.5)
Missing	33
Medication taken to 12 weeks of pregnancy with placebo	
Yes	123 (42.6)
Maybe	63 (21.8)
No	69 (23.9)
Missing	34

Among women who indicated they would participate in a planned trial that did not employ a placebo, the addition of a placebo did not significantly change their willingness to participate (47.4% without a placebo vs 48.8% with a placebo). The most common reason for hesitancy in willingness to participate or declining participation in the trial with a placebo, was only wanting the active medication (48.4%). Other reasons included excessive duration of medication if placebo (10.4%); too many medications if placebo (9.2%); concerns about the safety of medications in pregnancy (8.4%); a desire for more information (3.2%); and the emotional toll due to uncertainty of allocation (2.0%).

When restricted to women with three previous embryo transfers, a common definition of RIF, rates of agreement to participate were noted to be similar to all women (Table S1).

### Minimum clinically important difference (MCID) in fertility setting

A range of MCID values was noted within responding clinicians (minimum value 0%, maximum 40%), as well as between clinicians (mean 5%; median 5%, IQR 5–10%) and IVF patients (mean 5.5%; median 5%, IQR 1–10%).

### Motivations to prescribe and attitudes toward add‐ons

Clinicians were asked to indicate on a series of Likert scales to what extent they agreed with statements regarding their motivations for prescription of add‐ons in general.

**Figure 1:**
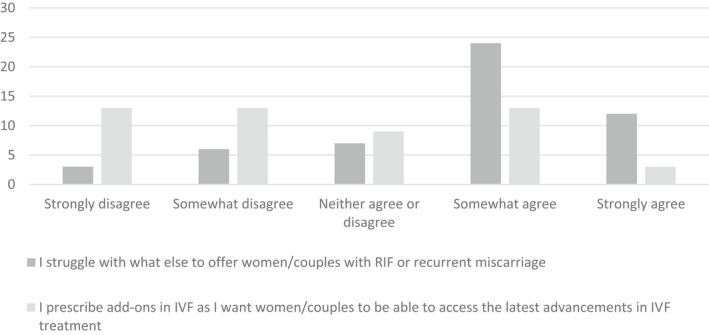
Clinicians' responses to questions regarding their motivations for prescription of add‐ons in *in vitro* fertilisation (IVF).

There were several reasons for offering add‐ons. Although most respondents agreed that add‐ons should only be offered once they have been proven to benefit couples (79%), many also thought it appropriate to offer such add‐ons as long as there was minimal risk of harm (59%). More than half of responding clinicians indicated they would prescribe an add‐on if requested to by a patient (Table S3). Many clinicians (69.2%) indicated they struggle with what else to offer couples with RIF (Fig. 1).

## DISCUSSION

The results of these surveys indicate that the Colorado Protocol is currently used in fertility care, and support the likelihood that patients would agree to take part in a clinical trial of this combined add‐on therapy. Almost half (48%) of surveyed IVF patients would participate if offered, and a further 25–30% would consider participating. The use of a placebo did not appear to deter IVF patients from trial participation, indicating a clinical trial of the Colorado Protocol under these conditions could be feasible.

These surveys provide data to support assumptions made in the planning of this RCT. However, wider use of the data is limited by the small numbers in each group, particularly the clinicians' survey. Additionally, the design of both surveys introduces the possibility of bias as those who chose to complete the questionnaire may be different from IVF patients in general. Another limitation was the lack of a clear denominator in either survey, precluding the reporting of a reliable response rate.

The survey of IVF patients was limited to New Zealand as this is the primary population from which the planned RCT will recruit. Respondents were asked if they would be willing to participate in the planned trial; however, it is unknown if this would truly translate into trial participation. Other studies suggest that survey responses do not always reflect patient behaviour with regard to patient participation in clinical trials.^7^

The addition of a placebo‐controlled design did not markedly alter IVF patients’ desires to participate with almost 80% indicating they would consider participating. These findings are reassuring and in agreement with other New Zealand surveys regarding participation in a hypothetical placebo‐controlled trial, where there was 59% agreement to participate among primary care patients,^8^ and 73% agreement (25% definitely, 48% maybe) among pregnant women.^9^ This proportion was significantly higher than other surveys in Japan where willingness to participate was only ~30%.^10^, ^11^ However, feasibility surveys for a placebo‐controlled endometriosis trial in the United States indicate a surprisingly high (90%) agreement to participate.^12^ These disparate findings may reflect the daily impacts of the condition being treated, and the perceived effectiveness of the treatment, but may also be influenced by cultural differences between the countries.

The MCID represents the smallest amount of benefit that a patient can recognise and value, and is emerging as a way to bridge the divide between statistical significance and clinical relevance. Assessments as to whether the benefits of a specific intervention are sufficient to offset its costs and inconveniences should primarily reflect patients' preferences. However, patients tend to regard even the smallest effect sizes as clinically important.^13^ Ideally, the proposed Colorado Protocol clinical trial should be powered to detect effects equivalent to or smaller than the median MCID reported here (5%), equating to approximately 2000 participants.

This data will be useful for the development of a protocol for a clinical trial of this combined add‐on therapy. The inclusion criteria for this planned RCT, specifically the criteria used to define RIF, may require further refinement, as significant heterogeneity was found in the definitions of this condition offered by surveyed clinicians. Consideration could be given to the use of a prediction model^14^, ^15^ taking into account the significant impact age has on the likely chance of success per cycle, rather than a single definition of the number of previous embryo transfers applied across all potential participants. The participant information sheet should include an explanation of the purpose of the placebo arm and sufficient information about medical safety to allay concerns. These steps will hopefully address the desire for more information among women who indicated they would be hesitant to participate.

## AUTHOR CONTRIBUTIONS

This research was conceptualised and instigated by CF. KA was the lead researcher. All authors contributed to the development of the content of the surveys. KA analysed the data. All authors contributed to this manuscript and approved the final version.

## Supporting information


**Appendix S1.** Clinician survey.
**Table S1**. Reasons for hesitancy to participate in a clinical trial under varying conditions among *in vitro* fertilisation patients.
**Table S2**. Agreement to participate in a clinical trial under varying conditions among *in vitro* fertilisation patients with three previous embryo transfer procedures.
**Table S3**. Clinicians’ attitudes toward prescription on add‐ons in *in vitro* fertilisation treatment.
