# Supplementary material for: Surveys of clinician and patient attitudes to an add‐on for in vitro fertilisation
Source: Aust N Z J Obstet Gynaecol. 2022 Jun 21;62(5):761–6. doi: 10.1111/ajo.13576 (PMC9796505; doi:10.1111/ajo.13576)
Supplement: Supplementary file 1 — Appendix S1. Clinician survey. Table S1. Reasons for hesitancy to participate in a clinical trial under varying conditions among in vitro fertilisation patients. Table S2. Agreement to participate in a clinical trial under varying conditions among in vitro fertilisation patients with three previous embryo transfer procedures. Table S3. Clinicians’ attitudes toward prescription on add‐ons in in vitro fertilisation treatment. [file AJO-62-761-s001.docx]

**Supplementary Appendix:**

Clinician survey

Haere Mai!  Welcome!

 Some common medicines are now being prescribed to aid fertility treatment, but we don’t yet have evidence about how helpful they are. We’re planning a new clinical trial to study the effectiveness of the Colorado Protocol during IVF treatment.

 The purpose of this survey is to ask doctors and nurses working at fertility clinics about their prescribing practices of add-ons in IVF treatment in order to understand how often these are used in clinical practice.

[ *Participant Information* ]

- I have read and understood this participant information and consent to participate in this survey

Are you involved in the care of women/couples undergoing IVF treatment?
*Ie. in a capacity as a doctor, or nurse employed at a fertility clinic in Australia, New Zealand or the United Kingdom*
*If you select 'No' you will directed to the conclusion of this survey as the following questions refer to your prescription or recommendation of add-on therapies for women/couples undergoing IVF treatment*

- Yes
- No

Skip To: End of Survey If Are you involved in the care of women/couples undergoing IVF treatment? Ie. in a capacity as a do... = No

Please select your clinical role from the list below

- Doctor
- Nurse
- Other ________________________________________________

IVF add-ons referred to in this survey are non-essential procedures, medicines, or laboratory techniques offered in addition to a woman/couples IVF treatment, with the aim to increase the chance of a live birth.

Are you aware of a package of IVF add-ons referred to as the “Colorado Protocol”?

- Yes
- No

Please select the medications you believe are part of the “Colorado Protocol” from the list below:*If 'other' please specify*

- Aspirin
- Heparin
- Prednisone
- Intravenous immunoglobulin
- Growth hormone
- DHEA
- Augmentin
- Doxycycline
- NSAIDs - ie. Ibuprofen
- Testosterone
- Antioxidants for male partner
- Antioxidants for female partner
- Other ________________________________________________

The "Colorado Protocol" package we refer to in this survey is comprised of the following add-on medications:
  
 ·       **Aspirin** Usually prescribed as 100mg orally daily, commenced from 1st day of ovarian stimulation in a fresh IVF cycle or 7 days prior to expected day 1 in an embryo-thaw cycle. Continued until at least the 13th week of pregnancy or until after pregnancy test confirming not pregnant.

·       **Prednisone** 
 Usually prescribed as 20mg orally daily, commencing the day after trigger injection in a fresh IVF cycle or 3-5 days before embryo transfer in an embryo-thaw cycle. Continued for 5 days only.

·       **Antibiotic**
 Usually Augmentin© (amoxicillin clavulanate) prescribed as 625mg orally twice daily, commencing the day after trigger injection in a fresh IVF cycle or 3-5 days before embryo transfer in an embryo-thaw cycle. Continued for 5 days only.

Within the past **1 year** have you recommended/prescribed the “Colorado Protocol” (aspirin, prednisone, and antibiotic in any dosages) for a woman/couple undergoing IVF treatment or a frozen embryo transfer?

- Yes
- No
- Prefer not to answer

Display if: If Within the past 1 year have you recommended/prescribed the “Colorado Protocol” (aspirin, predniso... != Yes

Within the past **1 year** how many times have you prescribed/recommended the  “Colorado Protocol” (aspirin, prednisone, and antibiotic) for a woman/couple undergoing IVF treatment or a frozen embryo transfer?

- 1-5 times
- 5-20 times
- 20+ times
- I have not recommended/prescribed the Colorado Protocol

Display if: Within the past 1 year have you recommended/prescribed the “Colorado Protocol” (aspirin, predniso... != No, Prefer not to answer

Within the past 1 year....

Have you recommended/prescribed Aspirin as an add-on for a woman/couple undergoing IVF treatment or a frozen embryo transfer?

- Yes
- No
- Prefer not to answer

Have you recommended/prescribed steroids (ie. Prednisone) as an add-on for a woman/couple undergoing IVF treatment or a frozen embryo transfer?

- Yes
- No
- Prefer not to answer

Have you recommended/prescribed antibiotics (Ie. Augmentin© or doxycycline) as an add-on for a woman/couple undergoing IVF treatment or a frozen embryo transfer?

- Yes
- No
- Prefer not to answer

Within the past **1 year** have you recommended/prescribed any of the following add-on therapies for a woman/couple undergoing IVF treatment or frozen embryo transfer?   
*If 'other' please specify medication, route, dosage, and frequency*

- Embryo glue
- Endometrial receptivity array (ERA)
- Endometrial scratch
- Heparin
- Growth hormone
- Androgens (testosterone)
- Melatonin
- IMSI
- PGT-A
- Time lapse monitoring
- Assisted hatching
- Ibuprofen (or other similar anti-inflammatory medication)
- DHEA
- Vitamins for female
- Vitamins for male
- Acupuncture
- Intrauterine hCG infusion
- Intralipid
- Platelet rich plasma
- Other ________________________________________________
- I have not prescribed any add-on therapies

For what indication/group of women/couples would you recommend the “Colorado Protocol” (aspirin, prednisone, and antibiotic)?

- Inherited thrombophilia
- Recurrent implantation failure (RIF)
- Male factor infertility
- Unexplained infertility
- High Natural Killer (NK) cell count
- Infertility due to endometriosis
- Recurrent pregnancy loss
- At my patient's request
- Other indication ________________________________________________
- All of my patients
- I never prescribe the "Colorado Protocol" to my patients

**The "Colorado Protocol" was initially developed as an add-on in IVF treatment for women/couples experiencing recurrent implantation failure (RIF).**

How do you define recurrent implantation failure (RIF) in your clinical practice?

________________________________________________________________

How effective do you believe the "Colorado Protocol" is at improving the number of live births from an IVF treatment or a frozen embryo transfer?

- Extremely effective ie. >10% increase - ie. increases the live birth rate from 15% to 25% per embryo transfer
- Very effective ie. 5-10% increase
- Moderately effective ie. 2-5% increase
- Slightly effective ie. 1% increase or less
- Not effective at all
- Don't know

What would be the minimum clinically important difference in live birth rate which would justify your prescription/ recommendation of an "add-on" in an IVF cycle/frozen embryo transfer?
 
eg. if 20% is the usual live birth rate after a single embryo transfer then how much higher much higher with the use of the Colorado protocol would this need to be ie. 5%, making a 25% live birth rate.
 
*Please specify the minimum value in the box below*

________________________________________________________________

The following statements refer to your views on IVF add-ons in general. 
Please indicate to what extent you agree with the statements below

|  | Strongly disagree | Somewhat disagree | Neither agree nor disagree | Somewhat agree | Strongly agree | Not applicable |
| --- | --- | --- | --- | --- | --- | --- |
| An add-on in IVF treatment should ONLY be offered to women/couples if there is evidence indicating benefit |  |  |  |  |  |  |
| I am more likely to prescribe an add-on in IVF treatment to a private patient than a public patient |  |  |  |  |  |  |
| I prescribe add-ons in IVF treatments if requested to by a woman/couple |  |  |  |  |  |  |
| Prescription of add-ons has no role in IVF treatment |  |  |  |  |  |  |
| Prescription of add-ons without an established evidence base in IVF is acceptable as long as there is no evidence of harm |  |  |  |  |  |  |
| I believe informing women/couples about the efficacy and safety of an add-on forms part of informed consent |  |  |  |  |  |  |

Please indicate to what extent you agree with the statements below

|  | Strongly disagree | Somewhat disagree | Neither agree nor disagree | Somewhat agree | Strongly agree | Not applicable |
| --- | --- | --- | --- | --- | --- | --- |
| Prescription of add-ons allows women/couples to feel like they are doing everything they can to achieve a pregnancy |  |  |  |  |  |  |
| Prescription of add-ons in IVF for women/couples with RIF gives them false hope |  |  |  |  |  |  |
| I struggle with what else to offer women/couples with RIF or recurrent miscarriage |  |  |  |  |  |  |
| Prescription of add-ons in IVF adds additional stress for women/couples to an already difficult process |  |  |  |  |  |  |
| I prescribe add-ons in IVF as I want women/couples to be able to access the latest advancements in IVF treatment |  |  |  |  |  |  |

**To conclude, please answer the following demographic questions.**
*If you feel that your response to these demographic questions would compromise the anonymity of your responses select 'Prefer not to say'.*

Please indicate your age bracket

- < 30 years
- 30-39 years
- 40-49 years
- 50-59 years
- 60+ years
- Prefer not to say

Please indicate your gender

- Male
- Female
- Non-binary / gender fluid
- Prefer not to say

Which ethnic group do you belong to? 
*Select those that apply to you. If 'other' please specify*

- NZ European/ Australian European/ Other European
- Māori
- Indigenous Australian, Aboriginal or Torres Strait Islander
- Pasifika
- Asian
- Middle Eastern
- Latin American
- African
- Other. Please state ________________________________________________
- Prefer not to say

In which country do you primarily practice?

- New Zealand
- Australia
- United Kingdom
- Other ________________________________________________

Are there any additional comments you would like to make about the topics included in this survey?  ____________________________________________________________

**By clicking "Submit" below, you are consenting to participate in this study.**
**Please be aware, as this survey is anonymous, your answers cannot be withdrawn once you have submitted them.**

Would you like to receive a summary of the survey results?
*If you select 'yes' you will be redirected to another survey to enter your contact details in order to maintain the anonymity of your responses*

- Yes
- No

IVF Consumer survey

Haere Mai!

   Some common medicines are now being prescribed to aid fertility treatment, but we don’t yet have evidence about how helpful they are. We’re planning a new clinical trial to study the effectiveness of aspirin, antibiotics and steroids during IVF treatment.   The purpose of this survey is to ask you about how you would feel about some things we might ask consumers to do when taking part in clinical trial research, for example, taking extra medicines during fertility treatment.

[ *Participant Information* ]

- I have read and understood this participant information and consent to participate in this survey

How did you hear about this survey?

- Through a link from FertilityNZ
- My fertility clinic had a link on their social media
- My fertility clinic emailed the link
- Other ________________________________________________

How many previous embryo transfer procedures have you had?  
*(Including as part of an IVF cycle or as a frozen embryo transfer)*

- 0
- 1
- 2
- 3
- 4
- 5
- 6
- More than 6

Have you and your doctor ever discussed taking optional "add-on" medications to help increase your chances of having a baby? 
*If yes please specify if you can*

- Yes ________________________________________________
- No

What other therapies have you used during any fertility treatment in the past?

|  | Therapies recommended by my doctor | Therapies I received | Therapies I paid for on top of the cost of my IVF |
| --- | --- | --- | --- |
| Acupuncture |  |  |  |
| Vitamins for female |  |  |  |
| Vitamins for male |  |  |  |
| Naturopathic treatment |  |  |  |
| DHEA |  |  |  |
| Ibuprofen (or other similar anti-inflammatory medication) |  |  |  |
| PGT-A |  |  |  |
| Time lapse monitoring |  |  |  |
| Assisted hatching |  |  |  |
| Embryo glue |  |  |  |
| Endometrial receptivity array (ERA) |  |  |  |
| Endometrial scratch |  |  |  |
| Heparin |  |  |  |
| Growth hormone |  |  |  |
| Androgens (testosterone) |  |  |  |
| Melatonin |  |  |  |
| IMSI |  |  |  |
| Others (please specify) |  |  |  |

Do you have an allergy to penicillin?
*ie. I have been advised by my doctor that I cannot take penicillin based antibiotics in the future*

- Yes
- No
- Prefer not to answer

*About taking part in a trial...*
 **A clinical trial is a research study in which people test a treatment to determine how well it works at treating a condition. We would like to ask a few questions about your views towards what we may ask volunteers to do in a future clinical trial...**

If your doctor recommended you to participate in a clinical trial, would you be willing to take a combination of 3 tablets around the time of your IVF/embryo transfer treatment?
[*This timeline indicates when the extra medications would be taken*]

- Yes
- Maybe
- No

Display if: If If your doctor recommended you to participate in a clinical trial, would you be willing to take a... = Maybe, No

Why?

- The number of medications is too much
- I am worried about the safety of medications during fertility treatment
- I am allergic to one of the medications
- Other ________________________________________________

Would you be willing to take the three tablets knowing that there is a fifty/fifty chance that these are dummy pills/placebos? 
*Placebos are pills or tablets that look, feel and taste the same as the active tablets but contain no active medicine at all.*

- Yes
- Maybe
- No

Display if: If Would you be willing to take the three tablets knowing that there is a fifty/fifty chance that th... = Maybe, No

Why?

- I would only want to take the active medication
- The number of medications is too much if these were dummy pills
- Other ________________________________________________

Would you be willing to take a daily mini aspirin pill (100mg) throughout the first trimester (first 3 months) of pregnancy?

- Yes
- Maybe
- No

Display if: If Would you be willing to take a daily mini aspirin pill (100mg) throughout the first trimester (fi... = Maybe, No

Why?

- This is too long for me to take a medication
- I am worried about the safety of taking aspirin in pregnancy
- I am allergic to aspirin
- Other ________________________________________________

Would you be willing to take a daily mini aspirin pill (100mg) throughout the first trimester of pregnancy knowing that there is a fifty/fifty chance that it is a dummy pill/ placebo?
*A placebo is a pill or tablet that looks, feels and tastes the same as an active tablet but contain no active medicine at all.*

- Yes
- Maybe
- No

Display if: If Would you be willing to take a daily mini aspirin pill (100mg) throughout the first trimester of... = Mayb, No

Why?

- I would only want to take the active medication
- The length of time taking the medication is too long if these were dummy pills
- Other ________________________________________________

About 25 people out of 100 will have a baby with IVF treatment. 
What is the smallest difference in the chance of pregnancy you would be willing to take these extra tablets during your IVF cycle for?

- 10% difference (ie. 35 people out of 100 would have a baby with their IVF treatment instead of 25)
- 8% difference (ie. 33 people out of 100 would have a baby with their IVF treatment instead of 25)
- 5% difference (ie. 30 people out of 100 would have a baby with their IVF treatment instead of 25)
- 3% difference (ie. 28 people out of 100 would have a baby with their IVF treatment instead of 25)
- 1% difference (ie. 26 people out of 100 would have a baby with their IVF treatment instead of 25)

*Finally.....*
**In order to ensure we are collecting the views of a range of women we would like to ask what ethnic group do you belong to?**
*Please select those that apply to you*

- New Zealand European
- Māori
- Samoan
- Cook Island Māori
- Tongan
- Niuean
- Chinese
- Indian
- Other such as Dutch, Japanese, Tokelauan. Please state ________________________________________________
- Prefer not to say

By clicking "Submit" below, you are consenting to participate in this study. 
Please be aware, as this survey is anonymous, your answers cannot be withdrawn once you have submitted them.

Would you like to receive a summary of the survey results?
If you select 'yes' you will be redirected to a different page to enter your contact details in order to maintain the anonymity of your responses.

- Yes
- No

**Supplementary Tables:**

| **Supplementary Table 1. Reasons for hesitancy to participate in a clinical trial under varying conditions among IVF patients** | | |
| --- | --- | --- |
|  |  | n |
| Combination of 3 medications without placebo arm | |  |
|  | Allergy to one of the medications | 5 |
|  | Safety concerns | 84 |
|  | Too many medications | 12 |
|  | Not planning more treatment | 9 |
|  | Uncertainty | 5 |
|  | Requiring more information about participation | 12 |
| Combination of 3 medications with placebo arm | |  |
|  | Only want the active medications | 57 |
|  | Too many medications | 23 |
|  | Safety concerns | 10 |
|  | Cost treatment with opportunity “lost” due to placebo | 3 |
|  | Emotionally hard due to uncertainty of active vs placebo | 2 |
| Medication taken to 12 weeks of pregnancy without placebo arm | |  |
|  | Safety concerns | 89 |
|  | Allergy to medication | 1 |
|  | Intervention too long | 6 |
|  | Requiring more information about participation | 7 |
| Medication taken to 12 weeks of pregnancy with placebo | |  |
|  | Only want active medication | 64 |
|  | Intervention too long | 26 |
|  | Safety concerns | 11 |
|  | Emotionally hard due to uncertainty of active vs placebo | 3 |

| **Supplementary Table 2. Agreement to participate in a clinical trial under varying conditions among IVF patients with 3 previous embryo transfer procedures** | | |
| --- | --- | --- |
| **(n = 129)** | | |
| n % | | |
| Combination of 3 medications without placebo arm | | |
| Yes | 73 | (56.6) |
| Maybe | 39 | (30.2) |
| No | 11 | (8.5) |
| Missing | 6 |  |
| Combination of 3 medications with placebo arm | | |
| Yes | 75 | (58.1) |
| Maybe | 21 | (16.3) |
| No | 25 | (19.4) |
| Missing | 8 |  |
| Medication taken to 12 weeks of pregnancy without placebo arm | | |
| Yes | 88 | (68.2) |
| Maybe | 25 | (19.4) |
| No | 8 | (6.2) |
| Missing | 8 |  |
| Medication taken to 12 weeks of pregnancy with placebo | | |
| Yes | 71 | (53.0) |
| Maybe | 25 | (19.4) |
| No | 25 | (19.4) |
| Missing | 8 |  |

| **Supplementary Table 3. Clinicians’ attitudes toward prescription on add-ons in IVF treatment** | | | | | | |
| --- | --- | --- | --- | --- | --- | --- |
|  | **Strongly disagree** | **Somewhat agree** | **Neither agree nor disagree** | **Somewhat agree** | **Strongly agree** | **Total** |
| An add-on in IVF treatment should ONLY be offered to women/couples if there is evidence indicating benefit | 1 (1.9%) | 6 (11.3%) | 4 (7.5%) | 17 (32.1%) | 25 (47.2%) | 53 |
| I am more likely to prescribe an add-on in IVF treatment to a private patient than a public patient | 29 (60.4%) | 5 (10.4%) | 7 (14.6%) | 7 (14.6%) | 0 (0.0%) | 48 |
| I prescribe add-ons in IVF treatment if requested to by a woman/couple | 8 (15.7%) | 8 (15.7%) | 6 (11.8%) | 24 (47.1%) | 5 (9.8%) | 51 |
| Prescription of add-ons has no role in IVF treatment | 16 (30.8%) | 19 (36.5%) | 11 (25.2%) | 1 (1.9%) | 5 (9.6%) | 52 |
| Prescription of add-ons without an established evidence base in IVF is acceptable as long as there is no evidence of harm | 4 (7.8%) | 7 (13.7%) | 10 (19.6%) | 18 (35.3%) | 12 (23.5%) | 51 |
| I believe informing women/couples about the efficacy and safety of an add-on forms part of informed consent | 0 (0.0%) | 0 (0.0%) | 0 (0.0%) | 6 (12.8%) | 41 (87.2%) | 47 |
| Prescription of add-ons allows women/couples to feel like they are doing everything they can to achieve a pregnancy | 6 (11.3%) | 2 (3.8%) | 8 (15.1%) | 26 (49.1%) | 11 (20.8%) | 53 |
| Prescription of add-ons in IVF for women/coupes with RIF gives them false hope | 2 (3.9%) | 15 (28.9%) | 10 (19.2%) | 18 (34.6%) | 7 (13.5%) | 52 |
| I struggle with what else to offer women/couples with RIF or recurrent miscarriage | 3 (5.8%) | 6 (11.5%) | 7 (13.5%) | 24 (46.2%) | 12 (23.1%) | 52 |
| Prescription of add-ons in IVF adds additional stress for women/couples to an already difficult process | 4 (7.7%) | 14 (26.9%) | 9 (17.3%) | 20 (38.5%) | 5 (9.6%) | 52 |
| I prescribe add-ons in IVF as I want women to be able to access the latest advancements in IVF treatment | 13 (25.5%) | 13 (25.5%) | 9 (17.7%) | 13 (25.5%) | 3 (5.9%) | 51 |
